# Insufficient Iron Improves Pristane-Induced Lupus by Promoting Treg Cell Expansion

**DOI:** 10.3389/fimmu.2022.799331

**Published:** 2022-02-28

**Authors:** Xiaofei Gao, Yang Song, Shuang Lu, Longyuan Hu, Meiling Zheng, Sujie Jia, Ming Zhao

**Affiliations:** ^1^ Department of Dermatology, Second Xiangya Hospital, Central South University, Changsha, China; ^2^ Hunan Key Laboratory of Medical Epigenomics, Second Xiangya Hospital, Central South University, Changsha, China; ^3^ Research Unit of Key Technologies of Diagnosis and Treatment for Immune-related Skin Diseases, Chinese Academy of Medical Sciences, Changsha, China; ^4^ Clinical Medical Research Center of Major Skin Diseases and Skin Health of Hunan Province, Changsha, China; ^5^ Department of Pharmacy, The Third Xiangya Hospital, Central South University, Changsha, China

**Keywords:** iron, oxidative stress, ROS, treg cells, lupus

## Abstract

Trace element iron affects T cell biology, but the knowledge about the role of iron in regulating Treg cell expansion is limited. Treg cells play an important role in keeping peripheral T cell tolerance, increasing Treg cell expansion is a promising therapeutic method for SLE. Here we showed that iron deficiency promotes Treg cell expansion by reducing ROS accumulation, improving the disease progression of pristane-induced lupus. Increased oxidative stress inhibits Treg cell differentiation by inducing cell apoptosis. Our data suggest that altering iron metabolism promotes Treg cell expansion by preventing oxidation-induced cell death, which may provide a potential therapeutic strategy for SLE.

## Introduction

Systemic lupus erythematosus (SLE) is a chronic complex autoimmune disease characterized by the overproduction of autoantibodies and multiple organ damage. Self-tolerance breakdown and effector T cell over-activation play an important role in the development of SLE. Treg cells serve as a key regulator for keeping peripheral T cell tolerance by restricting T cell overactivation, proliferation, and cytokine production (1). However, Treg cell dysfunction and overactivated effector T cells that produce pro-inflammatory cytokines are observed in SLE patients (2–4). Depletion of Treg cells accelerates the disease progression of SLE (5), while restoring Treg cell expansion by low-dose IL-2 treatment reduces the disease activity of SLE patients (6, 7), suggesting that increasing Treg cell expansion *in vivo* can be an effective method for SLE therapy.

Trace element iron is critical for T cell biology. Upon stimulation, T cells upregulate transferrin protein receptors to import serum iron for cell activation and energy metabolism (8, 9). However, excessive iron is harmful to cell biology. Intracellular iron overload promotes the secretion of inflammatory cytokines IFN-γ and GM-CSF, which contributes to the development of experimental autoimmune encephalomyelitis (EAE) (10). Iron metabolism is closely related to oxidative stress. Intracellular ferrous iron incorporates enzymes to catalyze ROS production, such as lipid ROS, which further induces ferroptosis and various diseases (11). Furthermore, iron accumulation is detrimental to SLE development. Iron deposition in the kidney worsens the progression of lupus nephritis (LN), leading to increased disease activity of SLE, while reducing the renal accumulation by iron chelator or hepcidin in the kidney improves the progression of LN in mice (12, 13), suggesting that altering iron homeostasis may be a promising therapeutic method for SLE.

A basic level of ROS is required for many biological processes such as cell proliferation and differentiation. However, increased oxidative stress is harmful to cell biologies, such as inducing DNA mutations, iron-dependent lipid oxidization, and cell death. In lupus, increased oxidative stress contributes to Treg cell depletion, suggesting that relieving oxidative stress may be an effective therapeutic method by promoting the differentiation of Treg cells (14).

In this study, we investigated the role of insufficient iron in Treg cell expansion and the development of SLE. We found that low iron diet (LID) promoted the expansion of Treg cells and reshaped the Th17/Treg cell ratio, thereby improving the disease progression of pristane-induced lupus. Insufficient iron contributes to Treg cell differentiation by reducing the ROS accumulation, which promotes cell death in Treg cells. Overall, our data show that reducing intracellular iron supports Treg cell expansion in SLE, which suggested a promising therapeutic method for SLE.

## Materials and Methods

### Human Subjects

Healthy donors were recruited from medical staff at the Second Xiangya Hospital.

### Mice

C57BL/6 mice were purchased from Slack Company (shanghai, China). The mouse forage was purchased from Dyets, Inc (Wuxi, China). For the LID mouse model, 3 weeks old female C57BL/6 mice were fed with low iron diet (5mg/kg) for 5 weeks, age-matched female B6 mice fed with normal iron diet (ND, 50mg/kg) were served as the controls. The nutrient elements of LID forage were consistent with the ND group except for the content of trace element iron. All mice were maintained in specific pathogen-free conditions. After 5 weeks of LID treatment, the spleen and dLNs (isolated from the inguinal lymph nodes) were collected for flow cytometric analysis, and CD4^+^T cells were isolated from the spleen for qPCR analysis.

### Pristane-Induced Lupus Mouse Model

For the pristane-induced lupus mouse model, 8-weeks old female mice were i.p. injected with 500 μl pristane(Sigma, catalog P9622) and fed with ND or LID for 6 months. After 6 months of pristane stimulation, mice were sacrificed for analysis. The urine protein was detected by a colorimetric assay strip (URIT). The spleen and dLNs (isolated from the inguinal lymph nodes) were collected for flow cytometric analysis, and the renal tissue was fixed in formalin and embedded in paraffin for histological analysis. Serum was collected for auto-antibody analysis.

### Histology

Hematoxylin and eosin (H&E) staining were used to evaluate the morphological changes of the kidney. Based on the criterion of the previous study, renal damage can be scored from 0-3: 0, normal; 1, slight cell proliferation and cell infiltration; 2, mesangial proliferation and a lobular structure formation; 3, crescent formation with hyalinosis (15).

### Immunofluorescent Staining

To determine the immune complex deposition in the kidney, paraffin-embedded renal sections were incubated with anti-mouse C3 antibody (Abcam, catalog ab200999) for mouse C3 staining and anti-mouse IgG antibody (Abcam, catalog ab205724) for mouse IgG staining. Fluorescence was labeled using Opal 7-color Manual IHC Kit (Perkin Elmer, catalog NEL811001KT). The image was captured by Perkin Elmer and analyzed by the Mantra system. The deposition of C3 and IgG was evaluated by Mean Fluorescence Intensity (MFI) using Fiji software (16).

### Flow Cytometry

For surface marker, cells were incubated with fluorochrome-conjugated antibodies against surface markers at 4°C for 30 min in the dark. For cytokine, cells were stimulated with PMA and ionomycin with the addition of GolgiPlug (BD Biosciences, catalog 550583) at 37°C and 5% CO2 for 6hr. For intracellular markers, cells were fixed and permeabilized with Cytofix/Cytoperm TM Fixation/Permeabilization Solution Kit (BD Biosciences, catalog 554714) or Foxp3/Transcription Factor Staining Buffer Set (eBioscience, catalog 00-5523-00), and then incubated with fluorochrome-conjugated antibodies at 4°C for an additional 30 min. For ROS detection, cells were loaded with DCFH-DA probe (Beyotime, catalog S0033S) at 37°C for 30 min protected from the light and washed twice with 1×PBS. For cell apoptosis detection, Annexin V Apoptosis Detection Kit I (BD bioscience, catalog 556547) was used according to the manufacturer’s instructions. Annexin V^+^ PI^+^ cells were determined as apoptotic cells. For intracellular iron detection, cells were loaded with FerroOrange (DOJINDO, catalog F374) at 37°C for 30 min in the dark. The following antibodies were used: FITC anti-mouse CD4 (Biolegend, catalog 100406), PE anti-mouse CD25(Biolegend, catalog 102007), AF488 anti-mouse CD25 (Invitrogen, catalog 53-0251-82), APC anti-mouse FOXP3 (Invitrogen, catalog 17-4776-42), APCCY7 anti-mouse IL-17A (BD Pharmingen, catalog 560821), PERCP-CY5.5 anti-mouse IFN-γ (BD Pharmingen, catalog 560660), PERCP-CY5.5 anti-mouse TNF-α (Biolegend, catalog 506321), BV786 anti-mouse T-bet (BD Pharmingen, catalog 564141), PE anti-mouse RORγt (BD Pharmingen, catalog 562607), PECY7 anti-human CD4 (Biolegend, catalog 317414), APC anti-human CD127 (Invitrogen, catalog 17-1278-42), BB700 anti-human CD127 (BD Pharmingen, catalog 566398), APC anti-human CD25(BD Pharmingen, catalog 555434), PE anti-human CD25 (Invitrogen, catalog 12-0259-42), APC anti-human FOXP3 (Invitrogen, catalog 17-4776-42).

### ELISA

Serum was collected for autoantibody detection. For anti-dsDNA IgGs, mouse anti-dsDNA IgG ELISA Kit (Alpha Diagnostic International, catalog 5120) was used according to the manufacturer’s instructions.

### RT-qPCR

Total CD4^+^T cells were isolated from the spleen of mice by mouse CD4 microbeads (Miltenyi Biotec, catalog 130-117-043). TRIzol reagent (MRC, catalog TR118) was used to extract total RNA from cells. HiScript III All-in-one RT SuperMix (Vazyme, catalog R333) was used to generate cDNA according to the manufacturer’s instructions. ChamQ Universal SYBR qPCR Master Mix (Vazyme, catalog Q711) was used for selected gene amplification. The relative expression of selected genes was analyzed by ΔΔCt method, which normalized to the house-keeping gene β-actin. The following primers are used: β-actin: FP, GTGACGTTGACATCCGTAAAGA; RP, GCCGGACTCATCGTACTCC. Il2r: FP, TGGCAACACAGATGGAGGAAG; RP, ACAGCCGTTAGGTGAATGCT. Foxp3: FP, CCCCCTCTAGCAGTCCACTT; RP, AAGTTGCCGGGAGAGCTGAA. Tfrc: FP, TTCGCAGGCCAGTGCTAGG; RP, TACAAGGGAGTACCCCGACAG. Fth: FP, CAGACCGTGATGACTGGGAG; RP, TCAATGAAGTCACATAAGTGGGGA.

### *In Vitro* Human Treg Cell Differentiation and ROSUP Treatment

Naive CD4^+^T cells were isolated from the peripheral blood of healthy donors, and cultured in the presence of anti-CD3 2 μg/ml (Calbiochem, catalog 217570), and anti-CD28 1μg/ml (Calbiochem, catalog 217669). For Treg cell differentiation, recombinant human TGF-β1 5 ng/ml (R&D, catalog 240-B-002), and recombinant human IL-2 10 ng/ml (PeproTech, catalog 200-02-10). Cells were cultured under the Treg cell-polarized conditions supplemented with 10% FBS (HyClone) at 37°C and 5% CO2 for 72 hr. For ROS stimulation, cells were treated with ROSUP (50 ug/mL) (Beyotime, catalog S0033S-2) for 12 hr, and then harvested for subsequent experiments. For iron supplementation, cells were treated with Hemin 100 μM (Selleck, catalog S5645) for 72 hr. For ROS inhibitor rescue, SOD 300U (Beyotime, catalog S0087) was added into cells after 24 hr of Hemin treatment.

### Statistics

SPSS 25.0 software was used for statistical analysis. All statistical results were presented as the mean ± S.E.M. For normally distributed data, two-tailed Student’s t-test was used for evaluating the statistical differences between the two groups. For abnormally distributed data, two-tailed Mann-Whitney U-test was used for assessing the statistical differences between two groups. One-way analysis of variance (ANOVA) with relevant *post hoc* tests was used for multiple comparisons.

## Results

### Iron Deficiency Contributes to Treg Cell Expansion

**Figure 1:**
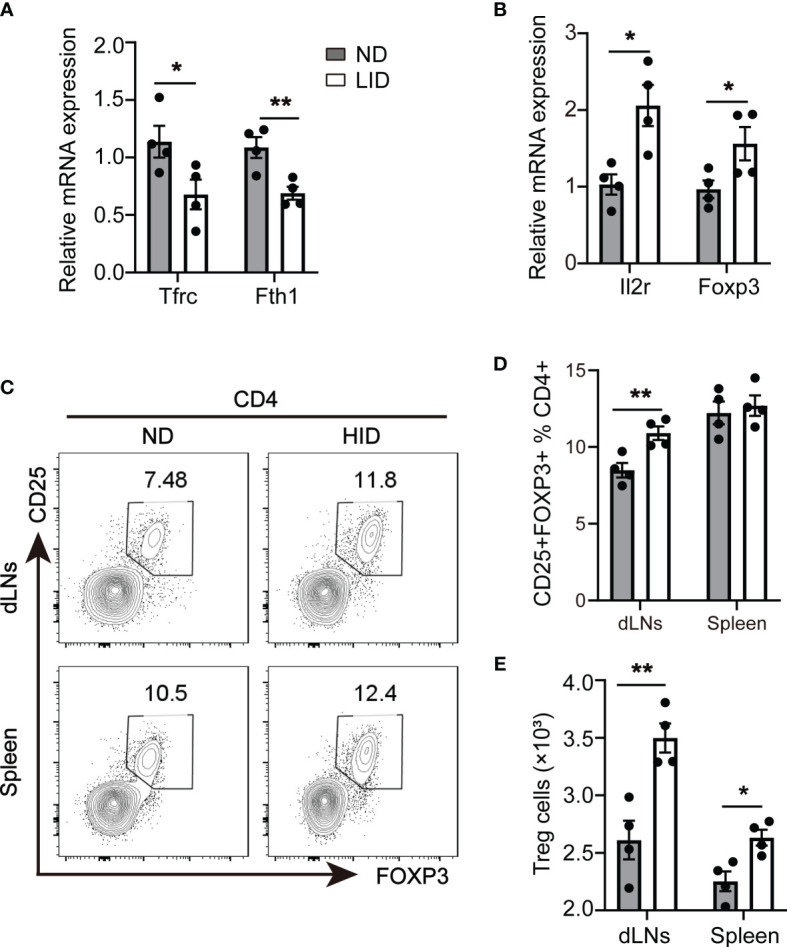
Insufficient iron contributes to Treg cell expansion. 3-weeks old female C57 mice were fed with ND or LID for 5 weeks and then were sacrificed for analysis. **(A, B)** qPCR of iron-related gene Tfrc and Fth1 **(A)** and Treg cell-related gene Il2r and Foxp3 **(B)** in splenic CD4^+^T cells of ND- and LID-treated mice. **(C)** Representative flow cytometry of Treg cells gated on CD4^+^T cells in dLNs and spleen of ND- and LID-treated mice. **(D, E)** quantification of the percentage and numbers of Treg cells in **(C).** **P < 0.01, *P < 0.05 (unpaired two-tailed Student’s *t*-test for **A**, **B**, **D**, **E**). Data are shown as mean ± S.E.M. Two independent experiments were performed, n = 4.

To explore the role of iron deficiency to Treg cell expansion, we treated 3-weeks old female c57/B6 mice with 5 mg/kg low iron diet (LID) for 5 weeks, mice treated with 50 mg/kg normal iron diet (ND) was served as the control group. After 5 weeks of treatment, the weight of LID-treated mice was slightly reduced compared with the ND group (**Supplementary Figure 1A**). We did not observe significant changes in the sizes of dLNs (including cervical, axillar, and inguinal lymph nodes) and spleen between the ND and LID groups (**Supplementary Figures 1B, C**). The percentage and number of total CD4^+^T cells were also similar between the two groups (**Supplementary Figures 2A, B**). Transferrin receptor (Tfrc) is responsible for cellular iron uptake (17). Fth encodes the ferritin heavy polypeptide 1 which controls intracellular iron storage when cells are in the iron-sufficient condition (18). To determine the intracellular iron homeostasis, we detected the expression of Tfrc (Transferrin receptor) and Fth 1 (Ferritin Heavy Chain 1) genes in the splenic CD4^+^T cells. As expected, the gene expression of Tfrc and Fth was significantly reduced in splenic CD4^+^T cells of LID-treated mice (**Figure 1A**). Next, we examined the changes of Treg cells after LID treatment. The expression of Treg cell-related genes Il2r and Foxp3 was significantly increased in splenic CD4^+^T cells of LID mice (**Figure 1B**). Consistent with the mRNA expression, the frequency of CD4^+^CD25^+^Foxp3^+^ Treg cells was significantly elevated in the dLNs (inguinal lymph nodes) of LID-treated mice compared with the ND controls, but it has no significant changes in the spleen (**Figures 1C, D**). However, the cell numbers of Treg cells were increased in both dLNs (inguinal lymph nodes) and spleen of LID-treated mice (**Figure 1E**). These results show that insufficient iron may contribute to Treg cell differentiation and expansion.

### Insufficient Iron Improves the Disease Progression of Pristane-Induced Lupus

**Figure 2:**
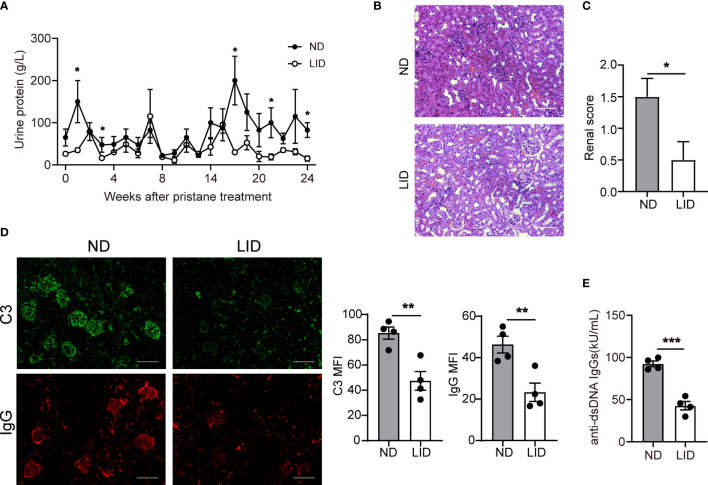
Insufficient iron improves the disease progression of pristane-induced lupus. 8-weeks old female mice were i.p. injected with 500 μl pristane and fed with ND or LID for 6 months. After 6-months of pristane stimulation, mice were sacrificed for analysis. **(A)** Urine protein levels of ND- and LID-treated mice. **(B)** Representative morphology of the renal tissues by H&E staining. Scale bar, 100 μm. **(C)** Histological scoring of the renal tissues in **(B). (D)** Representative immunofluorescent staining and quantification of C3 and IgG in kidney after 6-months of pristane stimulation. Green, C3; Red, IgG. Scale bar, 100 μm. **(E)** Serum level of anti-dsDNA IgGs after 6-months of pristane stimulation. ***P < 0.001, **P < 0.01, *P < 0.05 (unpaired two-tailed Mann-Whitney U tests for **A, C**; unpaired two-tailed Student’s *t*-test for **D, E**). Data are shown as mean ± S.E.M. Two independent experiments were performed, n = 4.

We assessed the disease progression of pristane-induced lupus in mice treated with ND or LID. 8-weeks old female C57/B6 mice were intraperitoneally injected with 500 μl pristane and fed with ND or LID for 6 months. Mice fed with 6 months of LID showed slight weight loss compared with the ND group (**Supplementary Figure 3A**). After 6 months of pristane stimulation, the urine protein level was reduced in mice treated with LID (**Figure 2A**). Morphological analysis also showed improved renal damage in LID-treated mice compared with the ND group (**Figures 2B, C**). Furthermore, the immune-complex deposition was reduced in the kidney of LID-treated mice (**Figure 2D**), and the titer of anti-dsDNA IgGs was also significantly decreased in mice fed with LID (**Figure 2E**). These results indicate that insufficient iron improves the disease progression of pristane-induced lupus.

### Insufficient iron Promotes Treg Cell Expansion in Pristane-Induced Lupus

**Figure 3:**
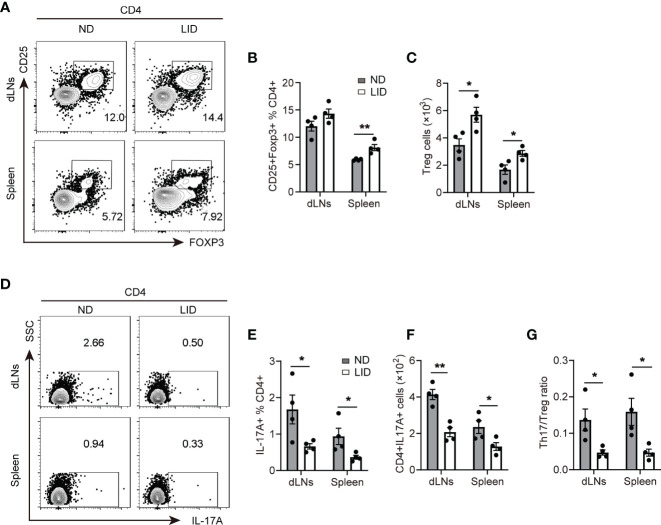
Insufficient iron promotes Treg cell expansion in pristane-induced lupus. 8-weeks old female mice were i.p. injected with 500 μl pristane and fed with ND or LID for 6 months. After 6-months of pristane stimulation, mice were sacrificed for analysis. **(A)** Representative flow cytometry of Treg cells gated CD4^+^T cells in dLNs and spleen of the ND- and LID-treated mice. **(B, C)** Quantification of the frequency and numbers of the Treg cells in **(A)**. **(D)** Representative flow cytometry of Th17 cells gated on CD4^+^T cells in dLNs and spleen of the ND- and LID-treated mice. **(E, F)** Quantification of the frequency and numbers of Th17 cells in **(D)**. **(G)** Quantification of the ratio of Th17 cells and Treg cells in **(B**, **E)**. **P < 0.01, *P < 0.05 (unpaired two-tailed Student’s *t*-test for **B, C**, **E–G**). Data are shown as mean ± S.E.M. Two independent experiments were performed, n = 4.

Since the defects in the differentiation and functions of Treg cells play an important role in the development of lupus, we asked whether insufficient iron can improve lupus by promoting Treg cell expansion. After 6 months of pristane stimulation, mice treated with LID showed an elevated percentage of Treg cells in the spleen (**Figures 3A, B**), and the cell numbers of Treg cells in the dLNs and spleen were increased (**Figure 3C**). To assess whether Treg cell expansion influence the Th17/Treg balance, which contributes to the disease progression of lupus, we determined the proportions of Th17 cells in pristane-treated mice fed with LID. We found that in the pristane-induced lupus mouse model, mice fed with LID showed the fewer frequency and number of CD4^+^ IL17A^+^ Th17 cells (**Figures 3D–F**) and the lower ratio of Th17/Treg cells compared with the ND controls (**Figure 3G**). These results suggest that insufficient iron promotes Treg cell expansion and reshapes the Th17/Treg cell balance in pristane-induced lupus-like diseases. In addition, we found that IFN-γ production was also reduced in the splenic CD4^+^T cells of LID-treated mice (**Supplementary Figure 4A**). However, the production of TNF-α and the expression of T-bet and RORγt were slightly reduced in LID-treated mice but without significant difference (**Supplementary Figures 4B-D**).

### Iron Deficiency Limits the Production of ROS in CD4^+^T Cells

**Figure 4:**
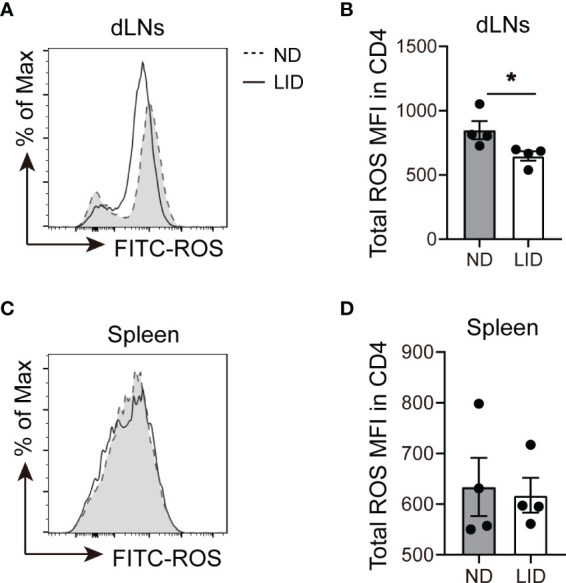
Iron deficiency limits the production of ROS in CD4^+^T cells. 3-weeks old female C57 mice were fed with ND or LID for 5 weeks and then were sacrificed for analysis. **(A, B)** Representative flow cytometry **(A)** and quantification **(B)** of total ROS in CD4^+^T cells of dLNs after 5-weeks of LID treatment. **(C, D)** Representative flow cytometry **(C)** and quantification **(D)** of total ROS in CD4^+^T cells of the spleen after 5-weeks of LID treatment. *P < 0.05 (unpaired two-tailed Student’s *t*-test for **B, D**). Data are shown as mean ± S.E.M. Two independent experiments were performed, n = 4.

Next, we sought to explore the mechanism of how iron deficiency affects the expansion of Treg cells. Previous studies have reported that iron promotes intracellular ROS production, which is harmful for Treg cell differentiation (11, 19, 20). Therefore, we determined the ROS levels in CD4^+^ T cells after LID treatment using DCFH probe (21, 22). We found that 5-weeks of LID treatment reduced the ROS levels in the CD4^+^ T cells of dLNs (**Figures 4A, B**), but there was no significant difference in the spleen (**Figures 4C, D**), which were consistent with the frequencies of Treg cells in the dLNs and spleen after 5 weeks of LID treatment (**Figure 1D**), indicating that Treg cell expansion is sensitive to intracellular ROS production.

### ROS Inhibits Treg Cell Differentiation by Promoting Apoptosis

**Figure 5:**
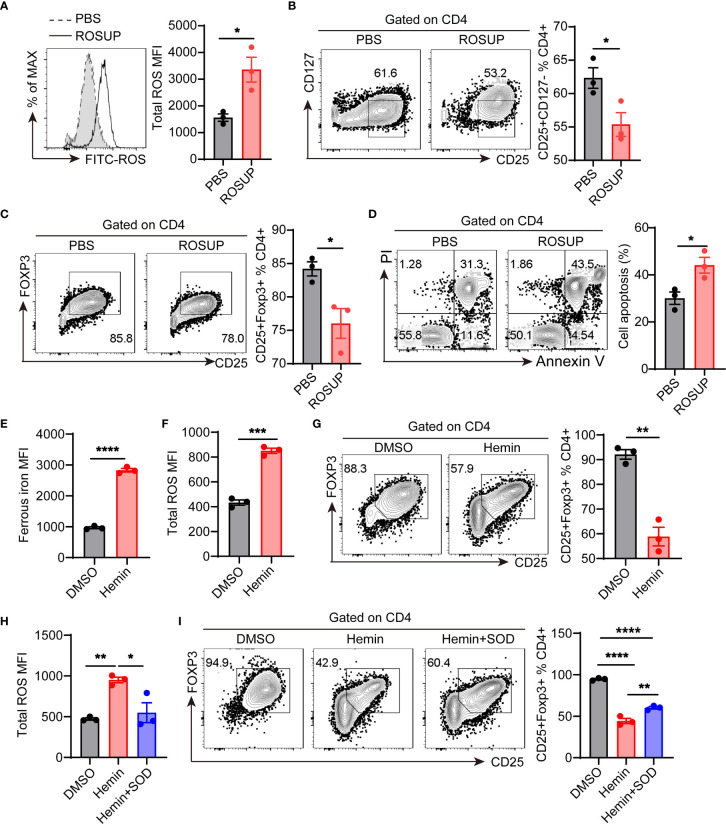
ROS inhibits Treg cell differentiation by promoting apoptosis. Human naive CD4^+^T cells were isolated from the peripheral blood of healthy donors and then cultured in Treg cell-polarized conditions with different treatments. After 3 days of Treg cell-polarization, cells were harvested for analysis. **(A)** Representative flow cytometry and quantification of total ROS in induced Treg cells after ROSUP treatment. **(B)** Representative flow cytometry and quantification of CD4^+^CD25^+^CD127^-^ Treg cells after ROSUP treatment. **(C)** Representative flow cytometry and quantification of CD4^+^CD25^+^FOXP3^+^ Treg cells after ROSUP treatment. **(D)** Representative flow cytometry and quantification of apoptosis in cells treated with ROSUP. Annexin V^+^PI^+^ is determined as apoptosis. **(E, F)** Quantification of ferrous iron **(E)** and ROS **(F)** in induced Treg cells treated with DMSO or Hemin. **(G)** Representative flow cytometry and quantification of CD4^+^CD25^+^FOXP3^+^ Treg cells in cells treated with DMSO or Hemin. **(H)** Quantification of ROS in induced Treg cells treated with DMSO, Hemin, or Hemin plus SOD. **(I)** Representative flow cytometry and quantification of CD4^+^CD25^+^FOXP3^+^ Treg cells in cells treated with DMSO, Hemin, or Hemin plus SOD. ****P < 0.0001, ***P < 0.001, ** P < 0.01, *P < 0.05 (unpaired two-tailed Student’s *t*-test for **A–G**; one-way ANOVA and Tukey’s multiple comparisons test for **H** and **I**). Data are shown as mean ± S.E.M. Two independent experiments were performed (n = 3).

To confirm whether ROS affects the differentiation of Treg cells, we used ROSUP to induce the overproduction of ROS during *in vitro* differentiation process of Treg cells. We isolated naive CD4^+^T cells from the peripheral blood mononuclear cells (PBMCs) of healthy donors. The cells were cultured in Treg cell-polarized conditions for 3 days and stimulated with ROSUP for 12 hr. ROSUP treatment significantly increased the level of ROS in induced Treg cells compared with the control group (**Figure 5A**). The percentage of Treg cells (gated by CD4^+^CD25^+^CD127^-^ and CD4^+^CD25^+^FOXP3^+^) was also significantly decreased in ROSUP treated group, which suggested that ROS impaired the differentiation of Treg cells (**Figures 5B, C**). ROS is harmful to cell biology, which can induce mitochondrial dysfunction and cell death. Therefore, we determined the cell apoptosis after ROSUP treatment. The result showed that ROSUP significantly elevated the frequency of cell death compared with the control group (**Figure 5D**). Next, we asked whether iron supplementation inhibits Treg cell differentiation by increasing ROS levels. FerroOrange was used to determine the levels of ferrous iron after iron supplementation treatment (23, 24). The results showed that iron supplementation by Hemin significantly increased the levels of ferrous iron and ROS in induced Treg cells (**Figures 5E, F**), and impaired the differentiation of Treg cells *in vitro* (**Figure 5G**). However, ROS inhibitor SOD rescued the defect of Treg cell differentiation in Hemin-treated cells by reducing ROS levels (**Figures 5H, I**). These data suggest that overproduction of ROS inhibits Treg cell differentiation by promoting cell apoptosis, and iron deficiency might promote Treg cell expansion by diminishing intracellular ROS accumulation.

## Discussion

Iron homeostasis plays an important role in immune cell biology, but the role of iron in regulating Treg cell expansion and function is far from clear. Defects in Treg cell differentiation and function promote autoimmune disorders, such as SLE (25). Th17/Treg cell imbalance was reported in SLE and positively correlated with the disease activity of SLE (26–28). Th17/Treg imbalance promotes autoimmune inflammation and organ damage in SLE, while altering the differentiation pattern of Th cells to reverse the imbalance of Th17/Treg cells promotes the disease remission of SLE (7, 27, 29, 30). Here we reported that insufficient iron promotes Treg cell differentiation. In the pristane-induced lupus mouse model, insufficient iron promoted the expansion of Treg cells and reshaped the Th17/Treg cell balance, leading to remission of renal damage and the lower level of autoimmune antibodies in pristane-induced lupus. Further study showed that iron deficiency inhibited the intracellular ROS accumulation which is harmful to Treg cell differentiation. These results demonstrate that altering the iron homeostasis reshapes the differentiation pattern of Th cells in autoimmune disorders, and intracellular iron status may be a key to regulating the metabolic characteristics and differentiation program of T cells.

Treg cell dysfunction contributes to effector T cell overactivation, which promotes autoimmune inflammation and the disease progression of SLE (25, 31). Therefore, expanding Treg cells and reconstituting the balance between the Treg cells and pathogenic Th cells provide a promising therapeutic strategy for SLE. However, the expanded Treg cells are short-lived and plastic, which may differentiate into pathogenic Th17 cells under the condition of IL-2 deficiency (32). Understanding the mechanism that regulates Treg cell expansion is needed to improve the effect of Treg cell-based therapy. We showed here that insufficient iron promotes the expansion of Treg cells, and reduces the Th17/Treg ratio in pristane-induced lupus, suggesting that intracellular iron homeostasis plays an important role in supporting Treg cell expansion.

ROS accumulation promotes abnormalities in Treg cell differentiation and function. Reducing ROS prevents Treg cell aging and inflammation, which improves the dysregulation of immune homeostasis (33). Inhibiting oxidative stress modulates the ratio of Th17/Treg cells, leading to the remission of psoriasis (34). Over-production of ROS can also kill T cells by oxidation (35, 36). Reducing intracellular ROS accumulation inhibits oxidative damage and the expression of apoptosis-related proteins P53 and Bax in splenic lymphocytes (37). Furthermore, ROS production of PBMCs is highly increased in SLE patients compared with healthy controls (38–40). ROS interferes with mTOR signaling to promote T cell overactivation in lupus, suggesting that ROS plays a pathogenic role in SLE development (41, 42). Our study demonstrates that insufficient iron promotes Treg cell expansion by reducing ROS accumulation in CD4^+^T cells, leading to the improvement of pristane-induced lupus. Furthermore, the increased ROS accumulation inhibits Treg cell differentiation by promoting cell apoptosis, suggesting that elevated oxidative stress may contribute to Treg cell dysfunction in SLE.

Together, our study shows that insufficient iron promotes Treg cell expansion by inhibiting ROS accumulation, improving the development of pristane-induced lupus. Our data suggest that keeping intracellular iron homeostasis is important for Treg cell differentiation. Furthermore, our study provides a possible mechanism that ROS over-production inhibits Treg cell differentiation by promoting cell apoptosis, which contributes to Treg cell dysfunction in SLE. Given that long-term iron deficiency may cause malnutrition and anemia, further study that specifically targets the iron metabolism in Treg cells is needed.

## Data Availability Statement

The original contributions presented in the study are included in the article/**Supplementary Material**. Further inquiries can be directed to the corresponding authors.

## Ethics Statement

The studies involving human participants were reviewed and approved by the Ethics Committee of the Second Xiangya Hospital of Central South University, China. The patients/participants provided their written informed consent to participate in this study. The animal study was reviewed and approved by the Institutional Animal Care and Use Committee (IACUC), The Second Xiangya Hospital, Central South University, China.

## Author Contributions

XG conceptualized the studies and wrote the manuscript. XG, YS, and SL analyzed the data and interpreted the results. XG, LH, and MLZ performed the experiments. SJ and MZ conceived the studies, interpreted the data, directed the studies, and revised the manuscript. All authors contributed to the article and approved the submitted version.

## Funding

This work was supported by the National Natural Science Foundation of China (No. 82030097 and 81874243), CAMS Innovation Fund for Medical Sciences (CIFMS) (2019-I2M-5-033), the Key project for international and regional cooperation in science and technology innovation of Hunan province (2019WK2081), the Project for leading talents in science and technology in Hunan province (2019RS3003).

## Conflict of Interest

The authors declare that the research was conducted in the absence of any commercial or financial relationships that could be construed as a potential conflict of interest.

## Publisher’s Note

All claims expressed in this article are solely those of the authors and do not necessarily represent those of their affiliated organizations, or those of the publisher, the editors and the reviewers. Any product that may be evaluated in this article, or claim that may be made by its manufacturer, is not guaranteed or endorsed by the publisher.
