# Supplementary material for: Insufficient Iron Improves Pristane-Induced Lupus by Promoting Treg Cell Expansion
Source: Front Immunol. 2022 Feb 28;13:799331. doi: 10.3389/fimmu.2022.799331 (PMC8918487; doi:10.3389/fimmu.2022.799331)
Supplement: Supplementary file 1 [file Datasheet_1.pdf]

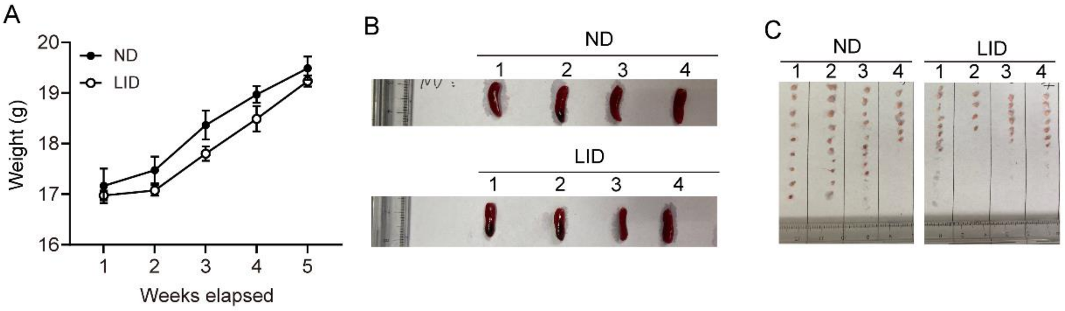

Supplemental Figure 1. Phenotypic observation of mice fed with LID. (**A**) Weight change of C57/B6 mice fed with ND or LID for 5 weeks (n=4). (**B** and **C**) Photos of spleen and dLNs after 5 weeks of LID treatment. For **A**, unpaired two-tailed Student's *t*-test was used. Data are shown as mean ± S.E.M.

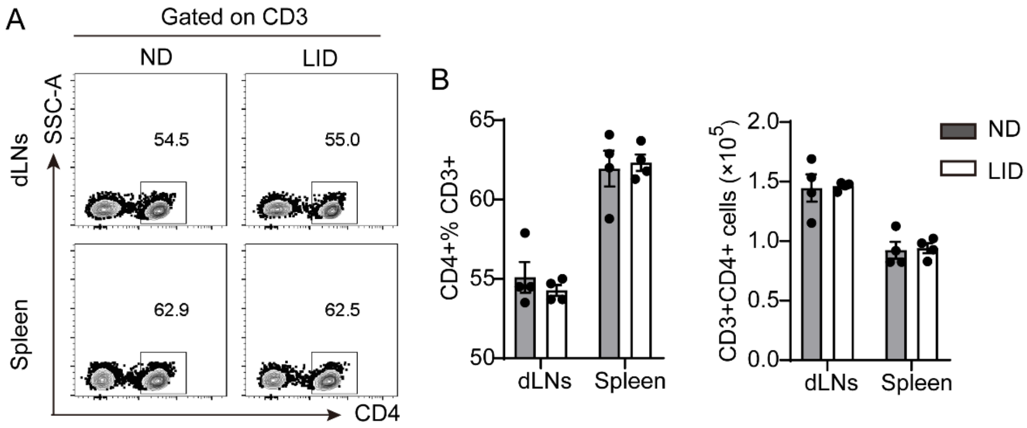

Supplemental Figure 2. The changes of total CD4$^+$T cells in mice fed with LID. (**A** and **B**) Representative flow cytometry (**A**) and quantification (**B**) of CD3$^+$CD4$^+$ T cells in dLNs and spleen of mice fed with ND or LID for 5 weeks. Data are shown as mean ± S.E.M. Unpaired two-tailed Student's *t*-test was used, n=4.

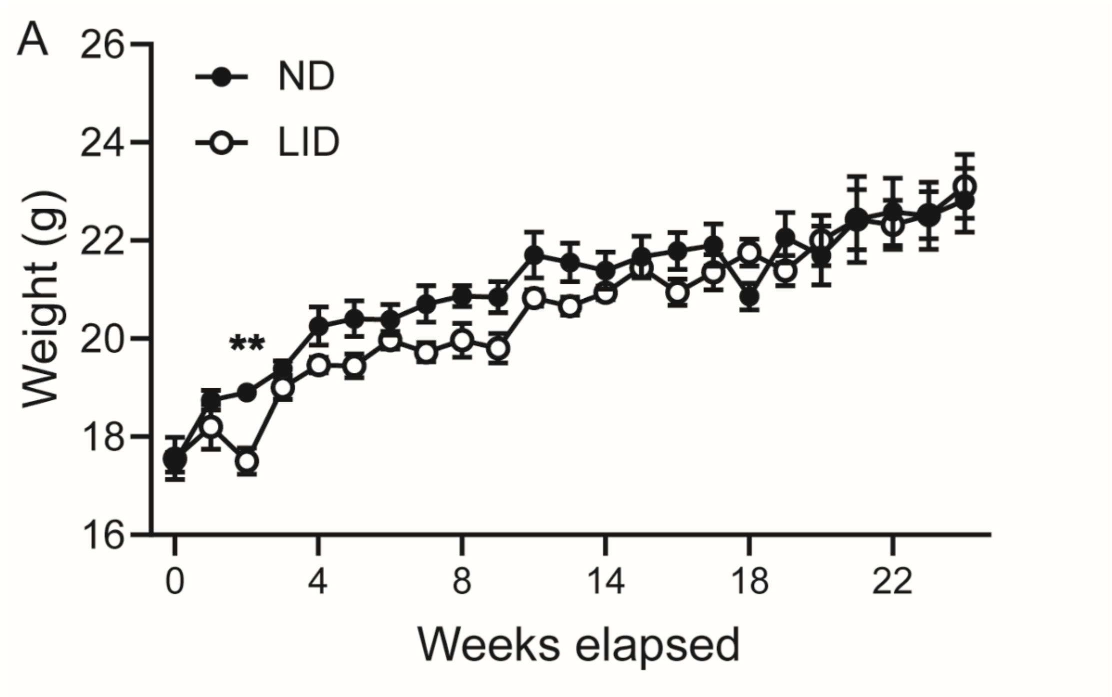

Supplemental Figure 3. Weight change of pristane treated mice. (**A**) Weight change of C57/B6 mice stimulated with pristane and fed with ND or LID for 24 weeks(n=4). **P < 0.01. Unpaired two-tailed Student's *t*-test was used. Data are shown as mean ± S.E.M.

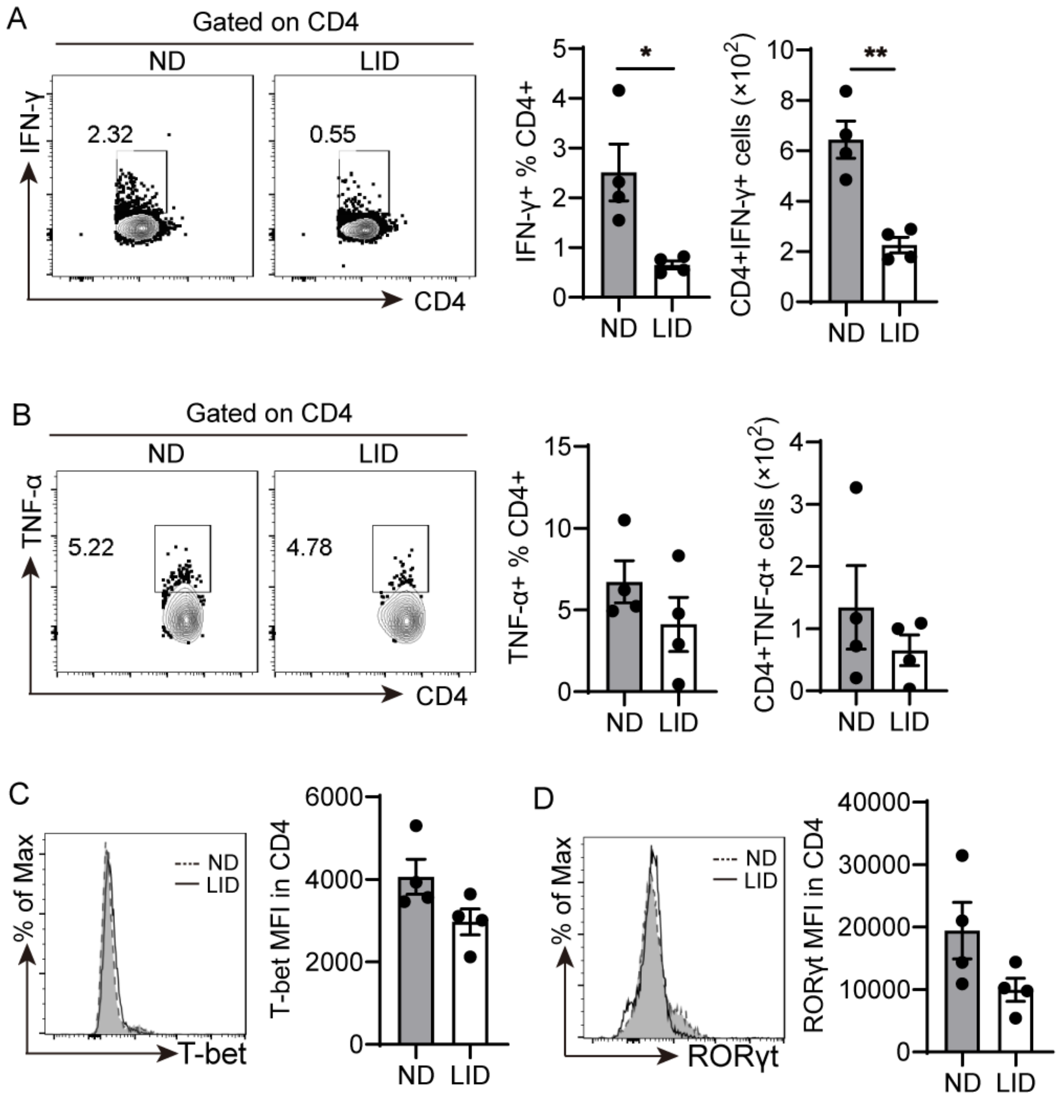

Supplemental Figure 4. Flow cytometry of Th1 and Th17-related genes in mice fed with LID in pristane mouse model. 3-weeks old female C57 mice were fed with ND or LID for 5 weeks, and then stimulated with pristane by intraperitoneal injection, after 7-months of pristane stimulation, mice were sacrificed for analysis. (**A**) Representative flow cytometry and quantification of IFN-γ in splenic $CD4^+$T cells of mice fed with ND or LID. (**B**) Representative flow cytometry and quantification of TNF-α in splenic $CD4^+$T cells of mice fed with ND or LID. (**C** and **D**) Representative flow cytometry and MFI quantification of T-bet (C) and RORγt (D) in splenic $CD4^+$T cells of mice fed with ND or LID. **P<0.01, *P<0.05 (unpaired two-tailed Student's *t*-test for **A, B**, **C**, **D**). Data are shown as mean ± S.E.M. Two independent experiments were performed (n=4).
